# Features of ENT Cases in the Emergency Department of a Tertiary Hospital in Greece: A Prospectively Driven Data Collection Study

**DOI:** 10.3390/healthcare11131943

**Published:** 2023-07-05

**Authors:** Nikolaos Papadopoulos, Emmanuel P. Prokopakis, Alexander D. Karatzanis, Manolis Linardakis, Evangelia Mourellou, Emmanouil K. Symvoulakis

**Affiliations:** 1 Clinic of Social and Family Medicine, School of Medicine, University of Crete, Voutes, 71003 Heraklion, Greece; linman@med.uoc.gr (M.L.); evangeliamourellou@gmail.com (E.M.); esymvoulakis@uoc.gr (E.K.S.); 2 Department of Otorhinolaryngology-Head and Neck Surgery, School of Medicine, University of Crete, 71003 Heraklion, Greece; eprokopakis@med.uoc.gr (E.P.P.); a.karatzanis@uoc.gr (A.D.K.)

**Keywords:** primary care, ENT, referrals, emergency department, urgent cases

## Abstract

Primary healthcare is the pillar of a well-functioning healthcare system. General practitioners (GPs) should have a broad skillset to cope with the various conditions they encounter in everyday practice. Ear, nose, and throat (ENT) cases are some of the most common reasons for seeking care. The study aimed to define the frequency and type of ENT disorders seen in the emergency department of a tertiary hospital in Greece. All patients examined by an ENT specialist in the emergency department setting, within a year, were recorded, as well as all referrals from private practice or primary care facilities. From September to December 2021, data were collected from patients who agreed to complete a two-minute survey, namely, the Generalized Anxiety Disorder Scale (GAD-2) questionnaire. During the study year, 4542 cases were documented, from which the most common conditions listed were external otitis (6.9%/314), epistaxis (6.7%/305), and impacted earwax (5.7%/261). The diagnoses that led to hospitalization were 336 and the most common were peritonsillar abscess (16.4%/55), epistaxis (8.0%/27), and facial nerve paralysis (7.4%/25). Referrals from GPs working in the public sector represented more than the half of the total. There was a significant correlation between an increased number of hospital visits and an increased GAD-2 score, in the semester before the current visit (*p* < 0.001). Referrals to ENT specialists represent around 5% of all cases examined, and about 8% of all visits required hospitalization. Interdisciplinary clinical and research investment into GP training is compulsory to regulate ENT referrals by GPs.

## 1. Introduction

Primary healthcare (PHC) is the pillar of a successful healthcare system. Many developed countries have taken action to fortify their PHC, not only in order to alleviate hospital burden, but also, as the World Health Organization stated, to provide integrated, constant and people-centered care [1]. As a result, general practitioners (GPs) were asked to broaden their competencies and knowledge to provide services to a greater variety of healthcare service seekers. Primary care services may be able to cover community health needs with capturing and monitoring procedures to assess implementation. Wensing et al. [2] supported the argument that a robust primary care system can be beneficial for both, health and economy, by providing prevention and management of chronic diseases and treatment of numerous common disorders. During the last decade, PHC in Greece, while still under long-lasting development, has achieved some progress. Studies assessing the academic needs of GP trainees, while suggesting new aspects and recommendations, have shown a promising disposition towards reforms [3]. New urban PHC units were created, and more GPs were hired to support new and pre-existing PHC units. The aforementioned PHC expansion aimed at delivering better primary health care to cover the needs of the patients while absorbing some of the burdening effects that emergency departments (ED) have been handling for decades. However, due to a variety of reasons, these reforms were implemented slower than expected. During the pandemic, the need to enhance primary care services became more evident since hospital access limitations led to a greater gap between care request and supply.

Ear, nose and throat (ENT) disorders are common reasons for seeking care in EDs. Patients are frequently referred to ENT specialists by GPs or other doctors. Sorichetti et al. [4] showed that ENT symptoms concern represent about 10% of all presenting cases in primary care. The explanation or the seriousness of these referrals remain a field of interdisciplinary review. ENT referrals tend to be arguable, as shown by Scott et al. [5], since with authors listed otorhinolaryngology as the second most ‘peculiar’ specialty for receiving referrals. The explanation for purposefully ‘weak’ referrals seems mainly to be inadequate training of GPs in ENT disorders. This is further supported by the studies of Hu et al. [6] and Sorichetti et al. [4], as the authors concluded that there was an insufficient degree of knowledge and skills in the otorhinolaryngology area among primary care professionals to uphold purposeful referrals. Furthermore, as Mayer et al. [7] showed in their 2020 study in the UK, there were few chances for medical students to be qualified in ENT morbidity, suggesting a necessity for a similar conversation at an undergraduate level. 

In Greece, the revised curriculum for the Family Medicine residency (2019) includes just a five-month training course in ENT, ophthalmology, urology, and orthopedics clinics combined [8]. Thus, GPs practicing at health centers are most likely not sufficiently trained to treat ENT disorders. Therefore, they refer some patients to hospitals for secondary care management without fully using their primary care tool potential. The aim of the study was to define the frequency and the type of ENT disorders seen in the emergency department of a tertiary hospital in Crete, Greece. A secondary aim was to assess patients’ anxiety levels.

## 2. Materials and Methods

In order to meet the goals of this study, all patients who sought care in the ED of the University Hospital of Heraklion, Crete, Greece, regarding ENT problems and were examined by an ENT specialist from September 2021 to August 2022 were included. Crete is the largest island of the country and is divided into four prefectures with a total population of 624,408 in the consensus of 2021. The prefecture of Heraklion is the most populous, with a population of 305,017, ranking 3rd in the country. The city of Heraklion has two hospitals (one tertiary and one secondary) that rotate shifts every 24 h. One of them is the University General Hospital (Pa.G.N.H), where the present study took place. The on-call shifts alternate every morning at 08:00.

The information obtained concerned the following: gender, age, date of visit, residence, the symptom for visiting the hospital, the final diagnosis, the outcome of the visit (admitted, patient refused hospitalization, and released after clinical examination with medical advice), and the number of hospital visits. Moreover, data were recorded regarding referrals from GPs or other health providers to ENT specialists in the ED. These data included information on whether the visit was due to a referral from a GP or other care provider and the rationale behind the referral. Also, the diagnoses were coded according to the International Classification of Diseases (ICD-10). However, total counts may differ due to some missing values.

In order to acquire this information, multiple database sheets were employed. The registry book of the ENT department provided most of the data. This registry documents every patient examined by an ENT specialist in the ED. In addition, an e-hospital register application provided information regarding demographic data, exams, and past visits to local hospital units. Moreover, further input derived from the patient’s individual per-visit card created in the ED and filled in by the doctor who examined the patient. These hardcopy cards were searched to offer additional or missing information.

Patients were conventionally divided into two groups according to the place of residence: urbanized (population more than 2000 inhabitants) and rural (population less than 2000 inhabitants). Population size limit was set based on the Greek law “Methodology of demarcated settlements”, where locations with less than 2000 inhabitants are considered as non-urbanized settlements [9]. From the data obtained, counts and hospital admission frequencies were extracted for each recorded diagnosis. Firstly, the diagnoses were coded according the International Classification of Diseases system ICD-10, grouping them with strict labeling. Afterwards, the cases admitted to the hospital were grouped considering the specific ICD-10 by the total cases of the same specific ICD-10 recorded to extract a Specific Hospitalization Index (SHI) [10]. During data collection, we noticed that some patients presented to ED with a feeling of a foreign body which was not confirmed by the medical examination. Due to the inability to list the following cases as a specific ICD-10, we conventionally added an X before the mentioned ICD-10. The visit’s outcome was categorized as (a) admitted, (b) patient refused hospitalization, and (c) released after clinical examination with medical advice.

Moreover, to assess the patients’ anxiety levels, adult attendees were later contacted via telephone within two weeks following their visit, from September to December 2021, to complete the Generalized Anxiety Disorder Scale (GAD-2) [11]. GAD-2 scale responses aimed to investigate its usefulness as a confounding determinant to indirectly influence seeking care motivation, especially when multiple visits or self-referrals occurred, GAD-2 has been previously used in Greek population, for research purposes, indicating significant relation to other scales measuring anxiety and depression, such as Patient Health Questionnaire-4 (PHQ-4) [12]. GAD-2 score consists of two questions, and each item is scored from 0 to 3. The questions are: a. ‘Over the last two weeks, how often have you felt nervous, anxious, or on edge?’, and b. ‘Over the last two weeks, how often have you been unable to stop or control your worrying?’. The given answers for both questions with their scoring must be one of the following: (a) Not at all (0), (b) Several days (1), (c) More than half the days (2), and (d) Almost every day (3). The sum of the two questions ranges from 0 to 6, with 0 being the lowest degree of anxiety and 6 the highest. The cut-off for a positive GAD-2 score is a minimum of 3. For this part of the study, the exclusion criterion implemented for the following questionnaire was the age under 18 years old. Out of 1454 cases, the number of patients eligible to complete the questionnaire was 1093. Of those patients, 700 were willing to complete the questionnaire. In total, 15.41% of all participants completed GAD-2. The ensuing scores were compared to gender, age, and the number of patients’ previous hospital visits.

To assess if referrals were purposeful, data were collected regarding the origin of the referral in correlation with the outcome of the visit. The sources of referrals were grouped into five categories: health centers, private practice of ENT specialists, private practice of GPs, other hospitals and public health care units, and other care involved professionals. Firstly, visit outcomes were used to determine whether the referral was rational. Secondly, the abovementioned groupings were utilized to evaluate the group with the most and least rational referrals. 

### Statistical Analysis

Data were analyzed using SPSS software, version 25.0 (IBM Corp). Frequency distributions of descriptive characteristics (demographic and clinical) were estimated for 4542 patients. Based on the ICD-10 classification, the most prevalent diseases and their explanations were presented. The relationship of referrals with the outcome of admission was assessed with the χ^2^ (Chi-squared) method and the difference in GAD-2 scores according to characteristics of patients and the number of visits with Mann–Whitney and Kruskal–Wallis tests. The Specific Hospitalization Index was also estimated [10] for the 20 most common diagnoses. Assessing monthly secular trends between the current study and the previous one, in 2006 [10], the number of visits was illustrated by implementing polynomial models as well as according to time (hour) distribution of visits for the current study.

This study was approved by the scientific committee of the University General Hospital of Heraklion (approval number 6371/07-07-2021) and from the Ethics committee of the University of Crete (approval number 59/05-05-2021, and it conforms to the principles of the Helsinki Declaration.

## 3. Results

**Figure 1:**
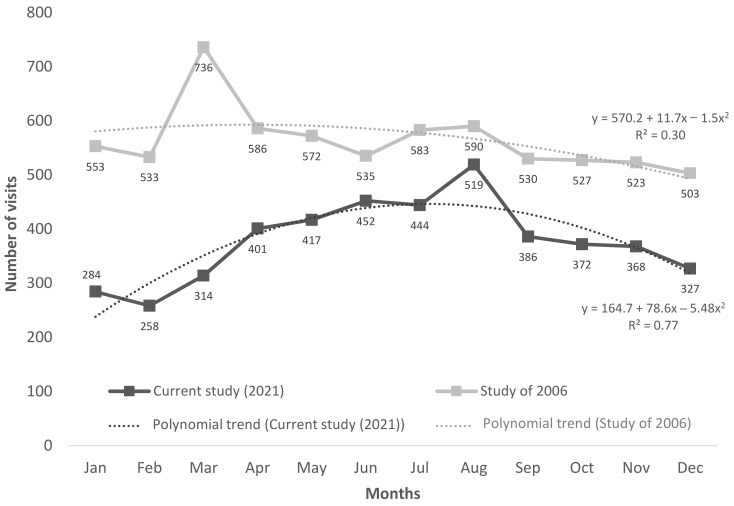
Comparative monthly distribution of the frequency and trend of visits to the ENT Department of a tertiary hospital in the years 2006 and 2021 (secular trend).

**Figure 2:**
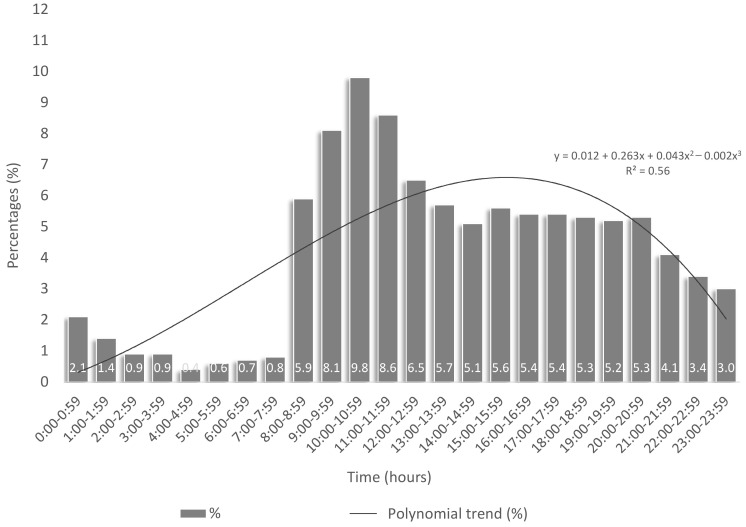
Hourly distribution of frequency and trend of 4461 visits to the ENT Department of a tertiary hospital over the duration of a year.

A total of 4542 patients sought care in the emergency department of the University Hospital of Heraklion and were examined by an ENT specialist for one year. Of those patients, 3938 were examined just once, and the rest (604) visited the ED for ENT problems multiple times. The ratio of men to women was almost 1:1, with 2325 men (51.6%) and 2182 women (48.4%). The median age of the patients examined was 44, in the range of 0–98 years old. The pediatric population was 623 cases or 13.7%. Most patients examined were living in urbanized regions and totaled 3267 (71.9%), compared to 1275 (28.1%) who resided in rural areas. The above is presented in Table 1. The seasonality of cases is illustrated in Figure 1. The month with the fewest patient visits was February, while the most visits were noted in August, with an overall increase during spring and summer months. The time of visit distribution can be seen in Figure 2. Most cases were listed during the day, with peak visits in the morning hours. Furthermore, there was no mortality observed in the ENT cases seen in the ED.

The twenty most common reasons for visiting an ENT specialist in the ED comprised 79.9% of the total cases recorded and are presented in Table 1. Forty-three other reasons for a visit were grouped into the remaining 20.1%. Earache and dizziness are the two symptoms that stand out with 902 (19.9%) and 449 (9.9%) cases recorded, respectively. Others are sore throat (325/7.2%), re-consultations of various reasons (315/6.9%), feeling of a foreign body (288/6.3%), and a first episode of nose bleeding (235/5.2%). The missing information comprised 286 cases (6.3%).

Additionally, 110 diagnoses were recorded and coded with the International Classification of Diseases system ICD-10 after grouping (Table 2). The missing values of this categorization represented 318 cases. The 20 most common ICD-10 diagnoses accounted for 73.7% of the cases. External otitis was the most common with 314 cases (6.9%). Epistaxis was second with 305 cases (6.7%), and impacted cerumen was third with 261 cases (5.7%). Dizziness unrelated to ENT morbidity accounted for 216 cases (4.8%) and was the 4th most common diagnosis. Acute otitis media accounted for 200 cases (4.4%) and acute pharyngitis for 163 (3.6%). We grouped the least frequent diagnosis in groups by the letter of the ICD-10. For the ICD-10 letter “H”: Eye and adnexa, ear and mastoid process (362 total diagnoses); the most frequent diagnosis was H68.1 (obstruction of the eustachian tube) with 61 cases (1.3%). For the letter “R”: Symptoms, signs, and abnormal clinical and lab findings (244 total cases); the most frequent diagnosis was R04.2 (hemoptysis), with 66 cases (1.5%). For the letter “J”: Diseases of the respiratory system (171 cases in total); the most common was J36 (peritonsillar abscess) with 61 cases (1.3%). Lastly, for the letter “K”: Diseases of the digestive system; the most common diagnosis was K11.2 (sialoadenitis) with 68 cases (1.5%). There was a difference in the number of cases with epistaxis recorded regarding the reason for visit and the ICD-10. The reason for that is that in the reason for visit, many cases of epistaxis were described as a re-consultation or a fall on the ground and hemoptysis.

Of the 4542 cases, 4236 had fully registered information (Table 3). Two hundred and nineteen cases (5.2%) of the above were referred to the ED to seek ENT specialist care. Furthermore, 29% of the referrals (65 cases) contributed to 19.3% of the total admissions (*p* < 0.001). At the same time, 5.9% of the non-referred cases contributed 80.7% of the total number of admissions. Of the 4236 cases, the number of patients admitted or suggested to be admitted to the hospital for in-patient care was 391 (9.23%). Seventy-one of these cases (18.1%) were referrals.

The total number of cases admitted to the hospital was 336 (7.3%). The 20 most common diagnoses admitted to the hospital are presented in Table 4. The peritonsillar abscess was the diagnosis with the most hospital admissions, accounting for 16.4% (n = 55). Epistaxis was the second most common cause of admission 8.0% (n = 27) and regarded almost exclusively cases of posterior epistaxis. Facial nerve paralysis accounted for 7.4% (n = 25) and sudden idiopathic hearing loss for 6.5% (n = 22). Cases of vestibular neuronitis and edema of the larynx accounted for 6.3% (n = 21) and 4.8% (n = 16), respectively. Subcutaneous head and neck abscesses accounted for 4.5% (n = 15). Pansinusitis and acute tonsilitis accounted for 3.3% (n = 11) each, while acute otitis media and unspecified edema (referring mainly to edema of the uvula) accounted for 3% (n = 10) each. Infectious mononucleosis contributed 2.7% (n = 9) of the total admissions. 

The results from the data collected from the 700 patients concerning the GAD-2 questionnaire are presented in Table 5 and Table 6. Women had higher mean levels in relation to men of GAD-2 score (2.07 vs. 1.84, *p* = 0.017) as well as the age group of 60–69 years in relation to 18–29 or 80+ (*p* < 0.001) (Table 5). Regarding the number of visits to the hospital, patients with a GAD-2 score 3+ had a higher mean number of visits in relation GAD-2 score of 0 (8.7 vs. 2.8 visits, *p* < 0.001). Moreover, 221 referrals were documented with two missing cases on the origin of the referral and outcome of visit (Table 7). GPs from health centers referred 131 cases (59.6%), and ENT specialists from private practice referred 25 cases (11.6%). GPs from private practice referred five cases (2.5%). Other specialists from private practice referred five cases (2.5%). Public hospitals and other public healthcare facilities referred 52 patients (23.8%). 

## 4. Discussion

ENT disorders are generally a common reason for seeking care in an ED. The attendance was almost the same for men and women, while most cases presented to the ED in the early morning hours and maintained a steady flow until the evening hours. The pediatric population accounted for 10% of the patients examined by an ENT specialist, while the age group 30–59 years old accounted for the majority of the visits. Almost a quarter of the cases recorded originated from a rural area around the city of Heraklion (28.1%). As expected, the most common symptom presenting to an ED pertaining to ENT examination was earache, while dizziness and sore throat followed. However, the most common triad of ENT diagnoses made was external otitis, epistaxis, and impacted cerumen. These are in accordance with the three most frequent urgent cases reported by van Bremen et al. [13] and Pino Rivero et al. [14], who published similar results in 2012 and 2005, respectively. Some interesting gender differences were found among studies implemented in Europe and India. Studies from Germany [15] and Spain [16] reported that female and male patients were almost equally included in both studies, likewise with the study onwards. However, two studies from India showed a male gender prevalence among care seekers, despite the different setting, with an urban [17] and rural [18] participant enrollment. It is not clear whether that difference occurred due to cultural, geographical, or other variables, but it should be taken into account when studying ENT cases attending EDs. Epistaxis was at the top three of the most common reasons of attendance in EDs, in all the aforementioned studies [15,16,17,18].

According to the 20 most admitted diagnoses and the extracted specific hospitalization index, the peritonsillar abscess, acute pansinusitis, vestibular neuronitis, mastoiditis, and edema of the larynx had the highest SHI. On the other hand, while being in the top 10 diagnoses recorded, epistaxis (n = 305), acute otitis media (n = 213), and acute tonsilitis (n = 142) had a very low SHI of 0.09, 0.05, and 0.08, respectively. This means that these conditions do not require focused in-depth care and can be treated as outpatient cases.

We were able to compare our findings with the epidemiologic study of Symvoulakis et al. [10] conducted in the same hospital. Our findings turned out to be quite different from the published study of 2006 [10]. The most common diagnoses then were acute tonsilitis 12.3%, acute pharyngitis 9%, and acute otitis media 7.6%. These differences mostly show some variation within service delivery due to the pandemic. Sore throat and fever, as symptoms, were the main manifestations of acute tonsilitis and acute pharyngitis, and patients with these conditions were mostly treated in the COVID-19 department of the ED. Nevertheless, acute otitis media, which often relates to earache, is most commonly seen by an ENT specialist. This may explain why the frequency of acute otitis media in our study (5.0%) remained close to the study of 2006 (7.6%). Moreover, the total number of patients recorded then was 6771, while currently, the number decreased to 4542. Additionally, there was an increase regarding the admissions, from 5.2% in 2006 to 7.4% in 2022. So, despite the 32.9% decrease in the total number of cases examined, the admissions increased by 2.2%. These findings are in coherence with the results of Fyntanidou et al. [19], which highlighted the crucial impact of the COVID-19 pandemic, as the leading cause of the reported variation. According to the results of their research [19], there was a decrease in attendance in the ED, with an increase in admissions, not only in ENT cases, but also in other specialties such as ophthalmology, cardiology, and general surgery. 

Taking into account that patients may have postponed seeking medical care during the COVID-19 period [20], it could be argued that a delayed appearance in the ED may relate to the increased severity of the cases and, thus to higher hospitalization rates. Less severe symptoms were rarely treated by a medical professional, or they were treated in the isolated COVID-19 setting of the hospital and were not examined by an ENT specialist, because of the resemblance of the symptoms (upper respiratory tract symptoms) to a COVID-19 infection. Overall, these reasons led to a decrease in the number of cases treated in the ED and probably altered the epidemiologic profile of ENT attendance, by decreasing totals of less severe cases and increasing the admission rate, as the denominator was reduced. Apart from that, less severe cases might be managed within PHC settings, and only cases with an increased likelihood of being admitted were actually referred to the tertiary hospital unit. A study in Lithuania showed a positive association between co-morbidity and referrals from PHC physicians [21]. To the authors’ knowledge, there was a lack of studies measuring the exact effect of PHC capacity on handling hospital referrals or admissions for ENT cases in Greece. However, a scoping review regarding PHC effectiveness in the country stressed that some improvement has been accomplished over the last years, although much effort is still needed [22]. Future research would be helpful to establish connections. 

Additionally, when comparing the seasonality of the two studies, there is a perceptible difference both in the polynomial curve of the studies and in the peak month. For example, in the study of 2006, the month with the most visitations was March, probably relating to allergic symptoms and upper respiratory tract infections [10]. However, in the current study, the peak month was August, with an increase in the spring and summer months. This could be attributed, as mentioned before, to the remarkable decrease in the number of visits to the ED due to COVID-19 for the local population throughout all year seasons. However, it is worth noting that Crete is a well-known travel destination, with the tourist season opening in early April and ending in early October. August of 2021 was registered as being highly booked, after the restrictions of the previous pandemic year, resulting in a temporary growth in the population of the island and, consequently, in more visitations to the ER. 

Diagnoses with the highest SHI, in the study of 2006 [10] and the current one, are similar but not overlapping. For example, peritonsillar abscess and edema of the larynx had two of the highest SHI in both studies. Moreover, Table 4 provides figures for the 20 most common diagnoses that were admitted to the hospital for inpatient treatment and their calculated SHI. Diagnoses with the highest SHI were mastoiditis (1.00), acute pansinusitis (0.916), and the peritonsillar abscess (0.901), with vestibular neuronitis (0.875) and Ramsay Hunt due to herpes zoster virus (0.857) accounting for the five most admission-listed diagnoses. However, epistaxis, acute otitis media, and acute tonsilitis had a very low SHI. These conditions rarely require inpatient treatment and can be managed within primary care settings if readily recognized and treated. 

More specifically, patients with peritonsillar abscess represented the most commonly admitted cases. The treatment requires primarily drainage of the pus either with needle aspiration or incision and then antibiotic treatment either orally or intravenously. The decision for outpatient treatment could be challenging and is rarely adopted as an option. If they are to be treated conservatively, patients need close monitoring and regular follow-up visits [23,24]. Mastoiditis requires inpatient treatment with IV antibiotic treatment because of the high frequency of complications in the case of conservative treatment [25]. Pansinusitis cases that led to admission occurred in children between 1 and 7 years of age. Due to the severe, albeit not very frequent, complications of pansinusitis in children, there is an inclination for inpatient treatment with intravenous antibiotics and monitoring [26], thus explaining the high SHI, as ENT specialists prefer inpatient treatment and close observation and monitoring of these cases. 

On the other hand, epistaxis rarely requires admission, as inpatient treatment is usually reserved for cases of posterior epistaxis. Most cases admitted were cases of posterior epistaxis, due to the difficulty in controlling the hemorrhage and the need for close airway monitoring [27]. Most cases of anterior epistaxis can easily be managed as outpatient cases with nasal packing and the use of topical vasoconstrictors such as oxymetazoline [28]. The treatment of acute tonsilitis cases usually includes conservative measures or, in some instances, antibiotics, depending on the Centor criteria, and it does not demand hospitalization unless complications appear [29]. Acute otitis media admissions in the current study mainly include children under the age of 10 years [30]. Since the management of these conditions rarely requires admission to the hospital or invasive techniques to treat them, it is safe to conclude that patients with these disorders can be safely treated in primary care settings by GPs.

A secondary observation of this study was to describe referral trends to the ED regarding ENT disorders by GPs and other medical professionals. While GPs from public primary units referred most of the patients (n = 132), the majority was treated as outpatients (83%; n = 110). All five patients referred by GPs working at a private practice were also treated and released with medical advice upon examination. In comparison, ENT specialists from private practice referred 25 patients, of which 72% (n = 18) were admitted to the hospital. These results indicate that GPs working either in public or private practice are most likely to refer patients to the ED for ENT disorders. It is important to mention that 50 of 132 cases referred from public primary units regarded cases of external otitis, acute otitis media, acute tonsilitis, and other conditions that require no specific equipment to diagnose or treat. One could think that GPs are uncertain in diagnosing and treating these conditions without the input of a specialist’s opinion, while ENT specialists from private practice tend to refer patients upon certainty of their diagnosis. Despite that, it could be argued that public PHC units usually receive a greater number of patients compared to other private clinics; therefore, it is possible to refer a higher number of cases. For example, Galanakos et al. (2023) reported 2617 median visits per month from a 24 h-operating PHC center with an ED in Athens [31]. They revealed that only 2.7% of the patients could not be treated within PHC environment and, thus, were referred to secondary or tertiary units, without estimating ENT morbidity [31]. However, these results may not be applicable in the whole country due to its peculiar geography and population density. In the literature, two other European studies revealed that PHC referrals were the second most common referral pathway of ENT cases presented in EDs after self-referrals, without dividing PHC physicians from public or private sector [15,16]

Furthermore, Wittchen et al. [32] showed that generalized anxiety disorder (GAD) is a common disorder met within primary care units. The anxiety levels of the attendees were assessed using the GAD-2 tool in correlation with age, gender, and number of visits to hospitals. In the current study, a sample of 1454 patients was examined in which the total number of patients who were able and willing and completed the questionnaire was 700, and the main result was the significant difference between genders. Studies by Vesga-Lopez et al. [33] and Wittchen et al. [34] also supported that women more commonly experience anxiety than men. As stated by Fisher et al. [35], men experience anxiety at high rates but are reluctant to seek help as they are often affected by gender-related attitudes and fear of stigma. Since the phone survey was performed days after the visit, in the range of two weeks, we cannot estimate the potential delay effect on GAD-2 scoring. However, it was also found that younger and older persons reported lower GAD-2 scores than middle-aged persons in the age range of 30–79 years. Kessler et al. [36] (2008) showed that the prevalence of GAD slightly differed depending on age. Patients in the age groups 18–29 and over 60 years developed lower levels of GAD than middle-aged persons aged 30–59 years. Furthermore, Santabárbara et al. [37] showed that anxiety increased in the general population by up to 3 times during the pandemic. It cannot be estimated if this external parameter in pandemic conditions could act as a group influencing factor beyond individuality, by altering variation in regard to GAD-2 scoring. A correlation emerged between GAD-2 scoring and the number of visits to hospital settings in the semester before the current visit, as the number of visits increased when the GAD-2 score increased from 0 to 3+. Patients with a positive GAD-2 score (3+) have a mean value of visits of 8.7/semester and a median of 5/semester. This significant relationship (*p* < 0.001) shows that GAD-2 levels and the frequency of visits to an ED evolve in parallel. Buccelletti et al. [38] found that panic attack disorders may lead to the somatization of symptoms, likely to contribute to ED over-attendance. Hudon et al. [39] showed that mental health disorders (including depression and anxiety) and chronic obstructive pulmonary disease were the single leading reasons for the high frequency of ED visits. Higher GAD-2 scores led to the excessive use of ED services, with longer waiting times and higher health care costs. Diagnosing and controlling anxiety disorders in the community should be prioritized by primary care physicians in an effort to alleviate both the psychological burden of the patients and facilitate better conditions with lower costs for the health system. Research focused on a well-designed large-scale, prospective study should be used to provide evidence on ENT epidemiology and related morbidity in order to enhance educational and clinical input, by taking into consideration local healthcare needs [40].

### Limitations of the Study

The present study has some limitations. Firstly, the data collected were from registries completed by doctors on duty, and it is possible that some information was of undersized or emphasized meaning. To minimize the missing information mainly regarding referrals, multiple sources of information for each patient visit were implemented. Secondly, out of 1093 eligible patients during the first four months of the study, 700 agreed to complete the GAD-2 questionnaire. Furthermore, current data were collected from one hospital setting and cannot be generalized or fully meet the needs of the community. Lastly, it cannot be distinguished whether the increased number of hospital visits was due to a high GAD-2 score or due to persisting symptoms that increased GAD-2 scoring and, in parallel, conditioned visit repetition in the previous semester. In other words, causation cannot be established. 

## 5. Conclusions

It is evident that the epidemiologic trends of ENT cases seen in the ED have changed during the pandemic. Fewer visits and more admissions than before were currently recorded. ENT emergencies tend to require assessment and treatment by ENT specialists, while less urgent ENT cases can be readily treated in a primary care environment. Most of the patients were self-referred. Referrals to ENT specialists represented around 5% of all cases examined. GAD levels relate to the number of hospital visits in the semester before the current visit, meaning there is a dependent relationship between the two variables. GPs may need more training during the residency in ENT morbidity in order to gain the necessary skillset to offer holistic services at the community. Clinical and research investment is educationally compulsory to standardize referrals by GPs, along with filtering unnecessary referrals and reducing self-referrals. To accomplish this, it is rational to extend and tailor-fit the training during general practice residency to current health needs, by opening evidence-based interdisciplinary dialogues, with synergistic benefits to patients and the community.

## Figures and Tables

**Table 1:** Descriptive characteristics of 4542 patients examined by ENTs in the emergency department in a tertiary hospital from September 2021 to August 2022.

		n	%
Gender	♂	2325	51.6
	♀	2182	48.4
Age, years	mean ± SD (median) [min, max]	43.8 ± 22.9 (44.0) [0, 98]	
	<12	405	9.0
	12–17	218	4.8
	18–29	765	16.9
	30–59	1870	41.4
	60–79	997	22.1
	80+	260	5.8
Revisits, number	1	3938	86.7
	2	420	9.2
	3+	184	4.1
Residencial Area	rural	1275	28.1
	urbanized	3267	71.9
Reason for Visit	Earache	902	19.9
	Dizziness	449	9.9
	Sore throat	325	7.2
	Feeling of foreign body	288	6.3
	First episode of epistaxis	235	5.2
	Head and neck pain	192	4.2
	Nasal congestion	128	2.8
	Head and neck trauma (except ear and nose)	124	2.7
	Hearing loss	104	2.3
	Fever	86	1.9
	Fall on the ground	80	1.8
	Hemoptysis	78	1.7
	Shortness of breath	76	1.7
	Nasal trauma	77	1.7
	Eyelid asymmetry	74	1.6
	Feeling of ear fullness	69	1.5
	Ear trauma	70	1.5
	Difficulty swallowing	56	1.2
	Tinnitus	42	0.9
	Revisit	315	6.9

**Table 2:** Classification and grouping of 4224 visits according to ICD-10 classification.

**ICD-10 Codes: 20 Most Prevalent Diseases and Explanations**			**n**	**%**
H60.9		External otitis	314	7.4
R04.0		Epistaxis	305	7.2
H61.2		Impacted cerumen	261	6.2
R42		Dizziness unrelated to ENT pathology	216	5.1
H66.9		Otitis media, unspecified	213	5.0
H81.1		Benign paroxysmal vertigo	200	4.7
J02		Acute pharyngitis	163	3.9
T16–18		Foreign body	157	3.7
J03		Acute tonsilitis	142	3.4
XT16–XT18		Feeling of foreign body	129	3.1
S00–S10		Head and neck trauma except nose and ear	120	2.8
S02.2		Fracture of nasal bones	113	2.7
S01.3		Open wound of ear	97	2.3
K07.6		Temporomandibular joint disorders	91	2.2
S00.3		Superficial injury of head	87	2.1
G51.0		Bell’s palsy	79	1.9
H62.2		Otitis externa in mycoses	71	1.7
L02.0, L02.1		Cutaneous abscess, furuncle, and carbuncle	73	1.7
J95.0		Tracheostomy complications	70	1.7
J01		Acute sinusitis	66	1.6
**Sum of the first 20 diagnoses**			**2967**	**70.2**
**ICD-10 Codes: Other Grouped Diseases and Explanations**			**n**	**%**
H		Eye and adnexa, ear and mastoid process	382	9.0
	H68.1	Obstruction of eustachian tube	61	13.5
R		Symptoms, signs and abnormal clinical and lab findings	234	5.5
	R04.2	Hemoptysis	66	14.6
J		Diseases of the respiratory system	171	4.0
	J36	Peritonsillar abscess	61	13.5
K		Diseases of the digestive system	143	3.4
	K11.2	Sialoadenitis	68	15.1
B		Certain infectious and parasitic diseases	79	1.9
	B27	Infectious mononucleosis	41	9.1
S & T		Injury, poisoning, and certain other consequences of external causes	77	1.8
	T78.4	Allergy, unspecified	43	9.5
L		Diseases of the skin and subcutaneous tissue	71	1.7
	L05	Pilonidal cyst	53	11.8
C & D		Neoplasm and diseases of the blood and blood-forming organs and certain disorders involving the immune system	39	0.9
Z		Factors influencing health status and contact with health services	21	0.5
	Z03.6	Observation for suspected toxic effect from ingested substance	21	4.7
XX		External causes of morbidity and mortality	11	0.3
	W01	Fall on the same level from slipping, tripping, and stumbling	10	2.2
I		Diseases of circulatory system	11	0.3
	I64	Stroke, not specified as hemorrhage or infarction	11	2.4
G		Diseases of the nervous system	8	0.2
	G50.0	Trigeminal neuralgia	7	1.6
F		Mental and behavioural disorders	6	0.2
	F41	Other anxiety disorders	6	1.3
Q		Congenital malformations, deformations, and chromosomal abnormalities	2	0.1
	Q38.5	Congenital malformations of palate, not classified elsewhere	1	0.2
E		Endocrine, nutritional, and metabolic diseases	2	0.1
	E06	Thyroiditis	2	0.4
**Sum of the rest of the cases**			**1257**	**29.8**
			451	100.0
**Total visits by classification**			**4224**	

**Table 3:** Relationship of referrals with the outcome of admission to the hospital for 4236 visits to the ENT Department of the ED of a tertiary hospital over the duration of a year.

	Referral					
	No		Yes		Totals	
	n	%	n	%	n	*p*-Value
Summary	4017	94.8	219	5.2	4236	–
Released with medical advice	3697	96.2	148	3.8	3845	<0.001
Admission	271	80.7	65	19.3	336	
Refusal of admittance	49	89.1	6	10.9	55	
	χ^2^ test.					

**Table 4:** The 20 most common diagnoses that were admitted to the hospital with the calculated Specific Hospitalization Index (SHI).

ICD-10 Codes: 20 Most Prevalent Diseases and Explanations		n of Admission	n of Total	%	SHI
J36	Peritonsillar abscess	55	61	90.1	0.901
R04.0	Epistaxis	27	305	8.8	0.088
G51.0	Facial nerve paralysis	25	79	31.6	0.316
H91.2	Sudden hearing loss	22	35	62.8	0.628
H81.2	Vestibular neuronitis	21	24	87.5	0.875
J38.4	Edema of the larynx	16	19	84.2	0.842
L02.0 & L02.1	Cutaneous abscess, furuncle, and carbuncle	15	73	20.5	0.205
J01.4	Acute pansinusitis	11	12	91.6	0.916
J03	Acute tonsilitis	11	142	7.7	0.077
H66.9	Acute otitis media	10	213	4.6	0.046
R60	Edema, unspecified	10	34	29.4	0.294
B27	Infectious mononucleosis	9	41	21.9	0.219
K11.2	Acute sieloadenitis	8	68	11.7	0.117
T16–T18	Foreign body	8	157	5.0	0.050
H61.0	Perichondritis of the external ear	7	20	35.0	0.350
S00–S10 ^a^	Head and neck trauma (except ear or nose)	7	120	5.8	0.058
B02.2	Zoster with other nervous system involvement	6	7	85.7	0.857
C & D	Neoplasms and diseases of the blood and blood-forming organs and certain disorders involving the immune system	6	37	16.2	0.162
H70.0	Mastoiditis and related conditions	6	6	100.0	1.000
J95.0	Tracheostomy malfunctions	6	70	8.5	0.085

^a^ without S00.3 S01.3 S02.2.

**Table 5:** GAD-2 score in terms of the characteristics of 700 visitors to the ENT Department of ED of a tertiary hospital from September to December 2021.

			GAD-2 Scale (Composite Score)		
		n	Mean Value	Median	*p-*Value
Summary		700	1.95	2.00	–
Gender	♂	355	1.84	2.00	0.017
	♀	345	2.07	2.00	
Age, years	18–29	138	1.08	1.00	
	30–39	100	1.97	2.00	
	40–49	111	2.26	2.00	
	50–59	98	2.11	2.00	<0.001
	60–69	115	2.34	2.00	
	70–79	94	2.30	2.00	
	80+	44	1.73	1.50	
Mann–Whitney and Kruskal–Wallis tests.					

**Table 6:** Number of visits to the hospital in terms of the GAD-2 score classification of 699 visitors to the ENT Department of the ED of a tertiary hospital over the duration of a year.

			Number of Visitations to Hospital Units of the Region of Crete in the Past Semester (Total 3642)			
		n (%)	Mean Value	Median	Summary	*p-*Value
GAD-2 Scale (composite score)	0	133 (19.0)	2.8	2.0	369	<0.001
	1	183 (26.3)	3.7	2.0	683	
	2	174 (24.9)	4.5	3.0	779	
	3+	209 (29.9)	8.7	5.0	1811	
Limit of 3+ is used for the diagnosis of general anxiety disorder (https://www.hiv.uw.edu/page/mental-health-screening/gad-2 accessed on 9 May 2023). Kruskal–Wallis test.						

**Table 7:** Relationship of referrals with the outcome of admission to the hospital in 219 visits to the ENT Department of the ED of a tertiary hospital as to the outcome.

	Outcome			
	Released with Medical Advice	Admission	Refusal of Admittance	
Referrals	n (%)			*p*-Value
Other Private Health Professionals	5 (100.0)	-	-	<0.001
Private General Practitioner	5 (100.0)	-	-	
Private Otorhinolaryngologist	7 (28.0)	18 (72.0)	-	
Public Primary Care Units	110 (83.3)	16 (12.1)	6 (4.6)	
Hospitals and other Public Practices	21 (40.4)	31 (59.6)	-	
χ^2^ test.				

## Data Availability

Data are available by the corresponding author upon reasonable request.
